# PM2.5 exposure reprograms cell cycle dynamics in uterine immune cells at single-cell resolution

**DOI:** 10.3389/fimmu.2025.1561290

**Published:** 2025-04-01

**Authors:** Lin Zhang, Jiaqi Tian, Yongfei Zheng, Shuyin Duan

**Affiliations:** ^1^ Clinical Medical Research Center for Women and Children Diseases, Key Laboratory of Birth Regulation and Control Technology of National Health Commission of China, Shandong Provincial Maternal and Child Health Care Hospital Affiliated to Qingdao University, Jinan, China; ^2^ Shandong Provincial Medical and Health Key Laboratory of Women’s Occupational Exposure and Fertility Preservation, Jinan, China; ^3^ Jinan (Preparatory) Key Laboratory of Women’s Diseases and Fertility Preservation, Jinan, China; ^4^ School of Public Health, Shandong First Medical University and Shandong Academy of Medical Sciences, Jinan, China

**Keywords:** fine particulate matter, cell cycle progression, immune cell heterogeneity, single-cell RNA sequencing, reproductive toxicity

## Abstract

**Background:**

Fine particulate matter (PM2.5) exposure has been associated with adverse effects on reproduction, yet the underlying cellular mechanisms remain poorly understood.

**Methods:**

Using single-cell RNA sequencing, we systematically investigated cell cycle dynamics of immune cell populations in the mouse uterus following PM2.5 exposure. Analysis of 9,000 balanced cells was performed to identify distinct cell populations and characterize changes in cell cycle distribution and gene expression profiles.

**Results:**

PM2.5 exposure induced distinct alterations in immune cell composition and cell cycle distributions. Notably, we observed significant changes in immune cell populations, including reductions in macrophages (510 to 58 cells), NK cells (445 to 91 cells), and granulocytes (1597 to 1 cells). Cell cycle analysis demonstrated cell type-specific responses to PM2.5 exposure: macrophages showed increased G1 phase representation (53.45%, +7.37%) with decreased G2M phase cells (18.97%, -12.79%), while NK cells exhibited relatively modest cell cycle alterations (G1: 28.6%, +2.5%; G2M: 45.1%, +2.6%; S: 26.4%, -5.1%). Differential gene expression analysis further identified crucial regulatory genes involved in cell cycle control, including Cd81 and Nrp1 in macrophages, Vps37b in NK cells. Integration of cell cycle markers with differentially expressed genes revealed distinctive phase-specific perturbations across immune cell types.

**Conclusion:**

PM2.5 exposure induces cell type-specific alterations in cell cycle progression of uterine immune cells, which provides novel mechanistic insights into environmental pollution-induced reproductive dysfunction.

## 1 Introduction

Air pollution, particularly exposure to fine particulate matter (PM2.5), has emerged as a significant environmental health concern with wide-ranging implications for human health (1). While the respiratory and cardiovascular effects of PM2.5 exposure have been extensively studied, growing evidence suggests that air pollution may also have profound impacts on reproductive health (2). Understanding the cellular and molecular mechanisms underlying these effects is crucial for developing targeted interventions and public health strategies.

The uterine environment represents a unique immunological niche, where precise regulation of immune cell function is essential for maintaining reproductive health and supporting successful pregnancy. Of which, the immune system in the uterus plays critical roles in tissue homeostasis, defense against pathogens, and regulation of the reproductive cycle (3). Any perturbation in this delicate balance may have significant consequences for reproductive function. Recent advances in single-cell RNA sequencing technology have revolutionized the current understanding of cellular heterogeneity and dynamic responses to environmental stimuli (4), and this powerful tool allows us to examine the effects of environmental exposures at unprecedented resolution, which reveals cell-type-specific responses and regulatory networks that may be masked in bulk tissue analyses.

PM2.5 exposure has been shown to induce oxidative stress and inflammatory responses in various tissues (5), but its specific effects on uterine immune cell populations remain poorly understood. Particularly important is the potential impact on cell cycle regulation and cellular senescence, as these processes are fundamental to maintaining healthy immune function and tissue homeostasis.

Cellular senescence is a state of permanent cell cycle arrest accompanied by distinct phenotypic changes, which has emerged as a critical factor in tissue aging and dysfunction (6). While senescence can serve protective functions, excessive accumulation of senescent cells can lead to chronic inflammation and tissue dysfunction. The potential role of PM2.5 exposure in inducing premature senescence in uterine immune cells represents an important gap in our understanding of environmental impacts on reproductive health. Of particular interest is the cell cycle regulation of immune cells, as proper immune cell cycling is crucial for maintaining tissue homeostasis and mounting appropriate immune responses (7). Disruption of immune cell cycle progression can lead to various pathological conditions, including impaired immune surveillance, altered inflammatory responses, and compromised tissue repair (8).

**Figure 1:**
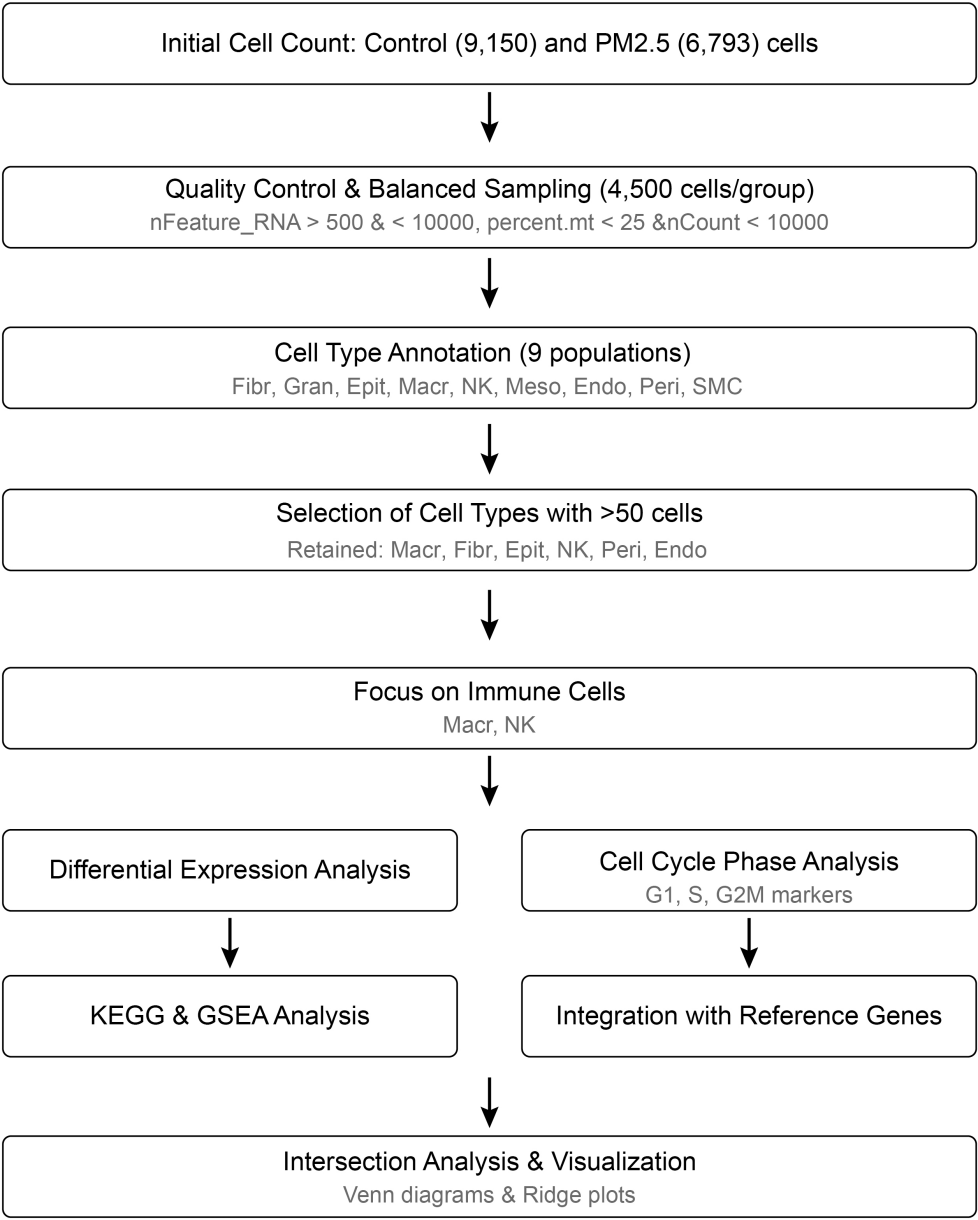
Flow chart of estrual immune cell targeted scRNA sequencing reanalysis.

To elucidate the effects of PM2.5 exposure on uterine immune cell populations, we reanalyzed our previously published single-cell RNA sequencing dataset with particular focus on cell cycle dynamics and senescence-associated changes (9). This dataset, generated from uterine tissues of PM2.5-exposed and control mice, was originally utilized to characterize cellular heterogeneity and reproductive toxicity. In the current study, we applied refined analytical workflows (**Figure 1**) to investigate cell cycle regulation, enabling the identification of phase-specific perturbations and senescence signatures across immune cell types. By integrating cell cycle markers with differentially expressed genes, we aimed to uncover novel mechanisms underlying PM2.5-induced immune dysfunction.

## 2 Methods

### 2.1 Animal PM2.5 exposure model

This study was approved by the Institutional Review Board of the Shandong Provincial Maternal and Child Health Care Hospital Affiliated to Qingdao University (NSFC.2022-023). For PM2.5 exposure, particles were collected from a major urban area using a TH-150 C Type III intelligent medium-flow air sampler, processed through lyophilization, and suspended in sterile saline. Female C57BL/6 mice (8 weeks old, Jinan Pengyue Experimental Animal Breeding Co., Ltd, Jinan, China) were housed under standardized conditions (12h light/dark cycle, 22 ± 1°C), and all animals were randomly divided into control and exposure groups (n=5 per group). The exposure group received intranasal inoculations of PM2.5 suspension (20 μL of 4.0 mg/mL) every other day for 6 days, while controls received equivalent volumes of saline. This administration protocol, adapted from our prior study (9), leverages the systemic translocation of inhaled PM2.5 to target organs, including the uterus, as evidenced by histopathological alterations and inflammatory marker induction. Although direct quantification of uterine PM2.5 levels was not performed here, the protocol’s efficacy in inducing uterine immune dysregulation has been previously validated.

### 2.2 Single-cell RNA sequencing

To prepare samples for sequencing, fresh uterine tissues were first enzymatically dissociated using collagenase type IV (1 mg/mL) at 37°C. Following digestion, single-cell suspensions were obtained by filtering through a 40 μm cell strainer, after which the cell concentration was adjusted to 1000 cells/μL while maintaining >90% viability. Once the cell suspensions were prepared, we proceeded with library construction using the 10× Genomics platform according to the manufacturer’s protocols. Subsequently, the libraries were sequenced on an Illumina HiSeq X platform, where we performed paired-end 150 bp sequencing with a target of 50,000 reads per cell to ensure adequate coverage.

### 2.3 Quality control and cell annotation

All analyses were performed using R (version 4.4.2). Key analysis packages included Seurat (v5.1.0) for single-cell analysis and clusterProfiler (v4.12.6) for pathway enrichment analysis. A complete list of package versions is provided in **Supplementary Table S1**. For initial data processing, raw sequencing data were first analyzed using Cell Ranger software and subsequently processed with the Seurat R package (10). To ensure data quality, we applied stringent filtering criteria, including nFeature_RNA values between 500-10000, mitochondrial gene percentage below 25%, and nCount_RNA less than 10000. Subsequently, we randomly selected a balanced set of 4500 cells from each group for downstream analysis. Following quality control, data were log-normalized using a scale factor of 10,000. Variable feature selection was performed using the “vst” method to identify 2,000 highly variable genes. Principal component analysis was conducted on these variable features. Based on elbow plot analysis, the first 8 principal components were selected for downstream analysis. Cell clustering was performed using the Louvain algorithm with a resolution parameter of 0.25. UMAP and t-SNE dimensionality reduction were performed using the same principal components.

Next, we performed cell annotation utilizing the Mouse Cell Atlas database, by which we analyzed the top 20 marker genes characteristic of each cell cluster to determine cell identities. A comprehensive list of cell type-specific marker genes used for annotation was provided in **Supplementary Table S2**. Finally, to ensure robust statistical analysis, we excluded any cell populations with fewer than 50 cells in either experimental group from subsequent investigations. Doublet detection was performed using DoubletFinder (v2.0.4) with parameters pN = 0.25 and pK = 0.09, identifying 548 potential doublets and 8,452 singlets. This additional quality control step was implemented to ensure robust cell type identification.

Additionally, batch effects between samples were corrected using the Harmony algorithm with default parameters. Integration efficacy was evaluated using silhouette scores and batch-corrected t-SNE plots (**Supplementary Figure S1**), demonstrating successful removal of technical variation while preserving biological differences.

### 2.4 Differential expression analysis

To identify changes in gene expression patterns, we conducted differential gene expression analysis using the Seurat FindMarkers function with default parameters. For rigorous statistical analysis, genes were considered differentially expressed only when they met both of the following criteria: an absolute log2 fold change of ≥ 1 and an adjusted p-value < 0.05. Since different immune cell types might respond distinctly to PM2.5 exposure, we performed this analysis separately for each of the identified immune populations, including macrophages and NK.

### 2.5 Functional enrichment analysis

For functional enrichment analysis, two complementary approaches were employed. First, enrichKEGG was applied to differentially expressed genes (DEGs; |log2FC| ≥ 1, adjusted p-value < 0.05) to identify pathways overrepresented in these gene sets relative to all expressed genes, which tested for statistical enrichment using a hypergeometric model. In contrast, gseKEGG was performed on genome-wide ranked gene lists (sorted by log2FC) to detect pathways with coordinated expression changes across all genes, including those with subtle but consistent trends, and this approach utilized a Kolmogorov-Smirnov-like statistic to assess enrichment at the extremes of the ranked list.

In detail, to understand the biological significance of the expression changes, we performed functional enrichment analysis using the clusterProfiler R package. First, we mapped the identified differentially expressed genes to KEGG pathways using the enrichKEGG function. Subsequently, we identified significantly enriched pathways by applying an adjusted p-value threshold of 0.05. Finally, to facilitate interpretation of these results, we generated comprehensive dot plots using custom R scripts, which simultaneously displayed both pathway significance levels and corresponding gene ratios.

To complement our pathway analysis, we performed Gene Set Enrichment Analysis (GSEA) using the gseKEGG function from clusterProfiler (11), while referencing the mouse Molecular Signatures Database. To ensure statistical robustness, we conducted the analysis with default number of permutations while maintaining default weight settings. After the analysis, we filtered the results for statistical significance using an adjusted p-value threshold of 0.05, then visualized the enrichment patterns using the gseaplot2 function to provide clear representation of the pathway dynamics.

### 2.6 Intersection analysis and visualization

To investigate the relationship between cell cycle regulation and differential gene expression, we conducted a systematic intersection analysis using the eulerr R package (12). In practice, we utilized two distinct sets of cell cycle markers: first, the established reference sets for S and G2M phases from the Seurat package (denoted as S.ref and G2M.ref). Second, a complementary set of phase-specific markers that we identified using the FindAllMarkers function within Seurat. After determining the intersecting genes between these sets and our differentially expressed genes, we visualized their expression patterns through ridge plots using the RidgePlot package. To comprehensively understand the cell type-specific responses to PM2.5 exposure, we performed analytical pipeline independently for each immune cell population, thereby enabling us to characterize distinct cell cycle perturbations across different immune cell types. Lastly, the significance of overlaps between DEGs and cell cycle phase markers was assessed using a hypergeometric test, which compared the observed overlap against a background of all expressed genes (n = ~12,000).

## 3 Results

### 3.1 PM2.5 exposure induces alterations in uterine immune cell populations

**Figure 2:**
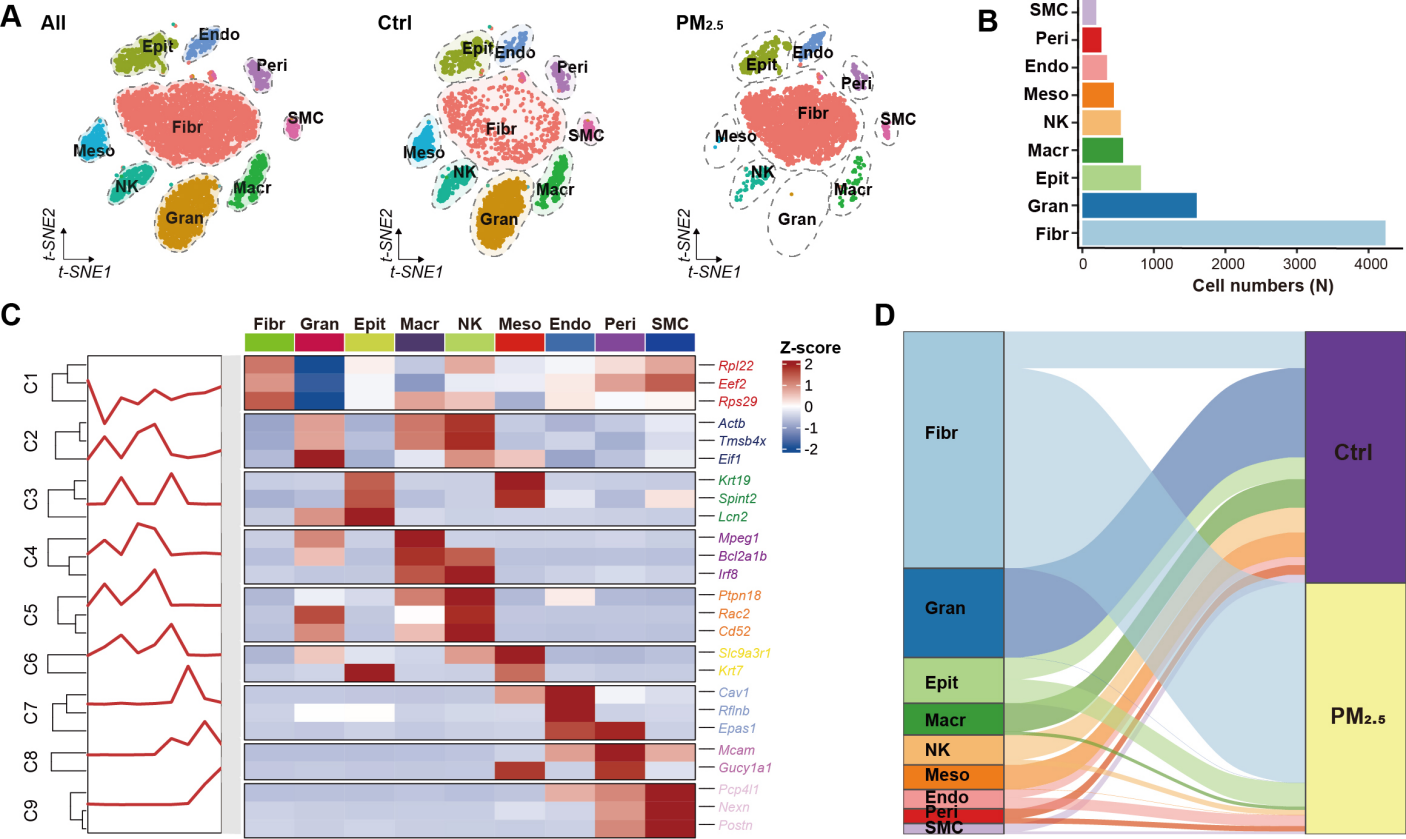
Uterine cell atlas revealed by single-cell RNA sequencing. **(A)** tSNE visualization of uterine cell clusters. **(B)** Bar plot showing the distribution of different cell populations. **(C)** Heatmap of cell type annotation based on top 3 marker genes for each cluster. **(D)** Sankey diagram illustrating cell distribution between different groups. Ctrl, control group; PM2.5, PM2.5 exposure group; Fibr, fibroblast; Gran, granulocyte; Epit, epithelial cell; Macr, macrophage; NK, natural killer cell; Meso, mesothelial cell; Endo, endothelial cell; Peri, pericyte; SMC, smooth muscle cell.

To investigate the effects of PM2.5 exposure on uterine immune cell populations, we performed single-cell RNA sequencing on uterine tissues from the normal control and PM2.5-exposed mice. Initial quality control analysis revealed 9,150 cells in the control group and 6,793 cells in the exposure group (**Figures 2A, B**). Following stringent quality filtering and balanced sampling, we obtained 4,500 cells per group for subsequent analyses. Cell type identification using canonical markers revealed 9 distinct cell populations, including immune cells (macrophages, NK cells, granulocytes), fibroblasts, smooth muscle cells, mesothelial cells, pericytes, endothelial cells, and epithelial cells (**Figure 2C**). Quantitative analysis demonstrated dramatic shifts in immune cell composition following PM2.5 exposure, with granulocytes showing the most pronounced change (1597 to 1 cells, p < 0.001), followed by significant reductions in macrophages (510 to 58 cells, p < 0.001) and NK cells (445 to 91 cells, p < 0.001) (**Figure 2D**).

### 3.2 Cell cycle phase analysis reveals cell type-specific responses to PM2.5 exposure

**Figure 3:**
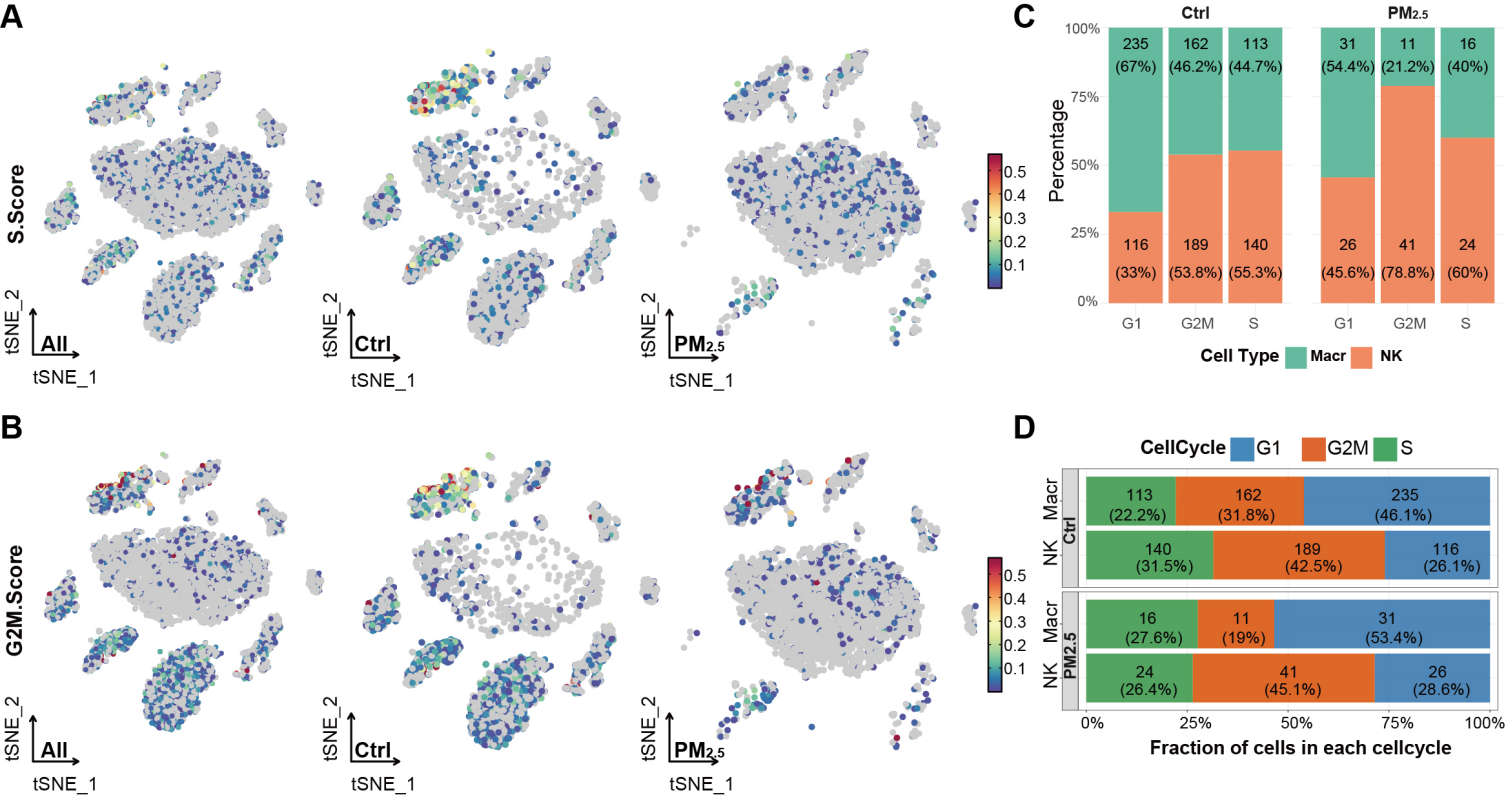
PM2.5 exposure disrupts cell cycles of uterine immune cells. **(A)** Scatter plot showing S phase scores across different uterine cell populations. **(B)** Scatter plot showing G2M phase scores across different uterine cell populations. **(C)** Stacked bar chart displaying the percentage distribution of cell cycle phases across different immune cell subpopulations between groups. **(D)** Stacked bar chart showing the proportion of cell cycle phases within each immune cell type across different groups. Ctrl, control group; PM2.5, PM2.5 exposure group; Macr, macrophage; NK, natural killer cell.

To elucidate the impact of PM2.5 exposure on cell cycle dynamics, we performed detailed cell cycle phase analysis across these two major immune cell populations. First, we calculated cell cycle scores based on the expression of phase-specific genes, where S and G2M scores were computed for each cell to determine its cell cycle state (**Figures 3A, B**). Macrophages exhibited a relatively balanced distribution with 46.08% in G1, 31.76% in G2M, and 22.16% in S phase, while NK cells showed a G2M-biased distribution (42.47% G2M, 31.46% S, 26.07% G1) (**Figure 3C**).

Following PM2.5 exposure, we observed moderate to significant alterations in cell cycle distributions. Macrophages exhibited G1 phase accumulation (53.45%, +7.37%) and reduced G2M representation (18.97%, -12.79%). In contrast, NK cells exhibited relatively modest cell cycle alterations, with G1 phase proportion increasing by 2.5% (28.6% total), a 2.6% rise in G2M phase (45.1%), accompanied by a 5.1% reduction in S phase cells (26.4%) (**Figure 3D**).

### 3.3 Differential gene expression analysis identifies distinct molecular signatures of PM2.5-induced immune cell dysfunction

**Figure 4:**
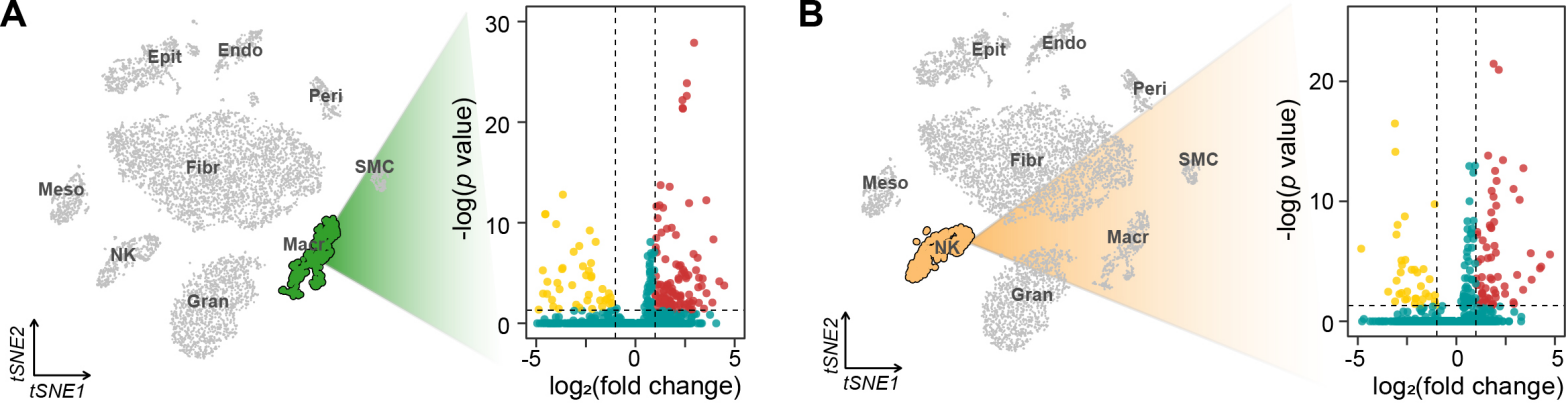
tSNE visualization and volcano plots of differentially expressed genes in macrophages **(A)** and NK cells **(B)**.

To understand the molecular mechanisms underlying alterations of different cell cycle phases, we performed comprehensive differential gene expression analysis for each immune cell type (**Figure 4**). Macrophages exhibited the most extensive transcriptional changes, with 160 differentially expressed genes (DEGs) (45 downregulated, 115 upregulated, FDR < 0.05). Among these, we identified significant upregulation of key regulatory genes including Cd81 (log2FC = 2.01, p.adj = 0.001), Nrp1 (log2FC = 1.58, p.adj = 0.002), and Cfh (log2FC = 1.42, p.adj = 0.024). NK cells showed more moderate transcriptional changes (100 DEGs: 37 downregulated, 63 upregulated), with notable alterations in cell cycle regulators such as Vps37b (log2FC = -2.75, p.adj < 0.001) and Ifitm1 (log2FC = 1.24, p.adj < 0.001).

**Figure 5:**
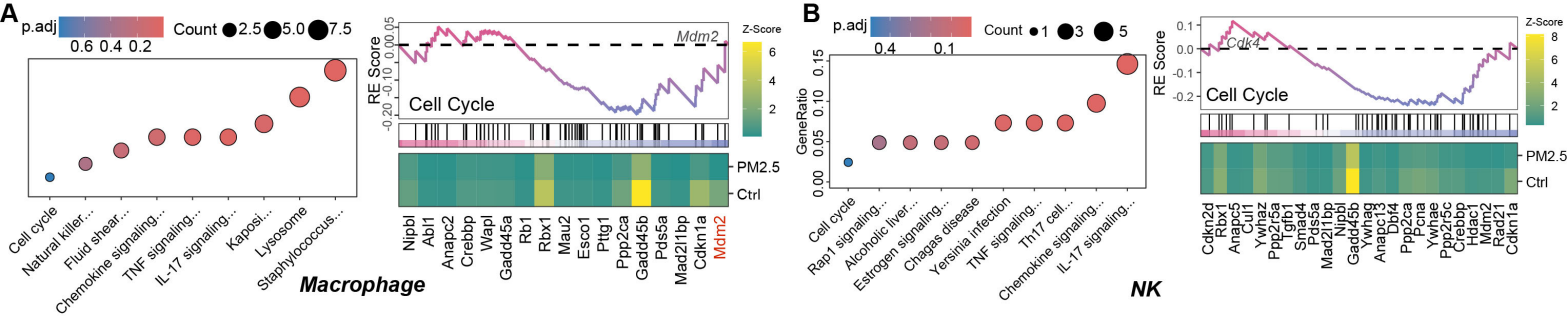
KEGG pathway enrichment analysis based on differentially expressed genes and gene set enrichment analysis (GSEA) based on genome-wide expression for macrophages **(A)** and NK cells **(B)**.

Key genes highlighted in the heatmap of GSEA plots in **Figure 5** were identified as dysregulated and enriched in the cell cycle pathway via enrichKEGG. Of these, downregulation of Mdm2 (log2FC = -3.87, p.adj = 0.035) and Cdkn1a (log2FC = -3.22, p.adj = 0.002) in macrophages reflects impaired p53 regulation and compensatory cell cycle alteration. In contrast, upregulation of Cdk4 (log2FC = 1.19, p.adj = 0.015) in NK cells underscores disrupted G1/S progression.

### 3.4 Integration of cell cycle marker genes reveals complex regulatory networks

**Figure 6:**
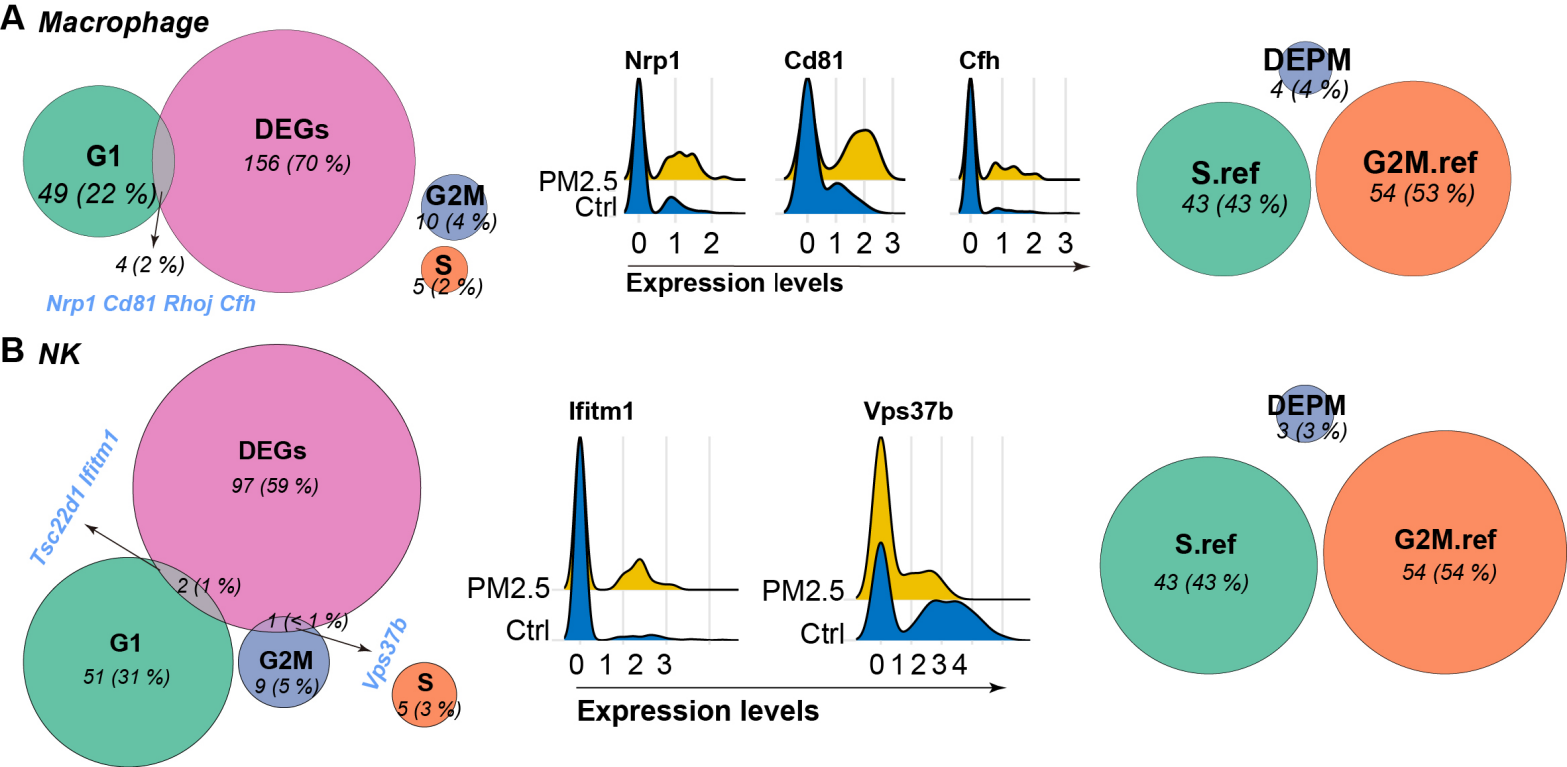
Venn diagram showing the overlap between differentially expressed genes (DEGs), established marker genes for G1, S, and G2M phases, differentially expressed phase marker (DEPM) genes, and reference genes for S and G2M phases derived from Seurat package for macrophages **(A)** and NK cells **(B)**. Expression of intersective items are visualized using Ridge plots between groups.

Further analysis on the expression of genes indicating different phases of cell cycle revealed distinct patterns across different immune cell populations (**Figure 6**). In macrophages, we identified significant overlap between DEGs and G1 phase markers, including Cd81, Nrp1, Rhoj, and Cfh, suggesting disruption of G1/S transition regulation. In parallel, NK cells showed specific alterations in G2M phase markers, particularly Vps37b, indicating potential impact on mitotic progression.

### 3.5 Cell type-specific senescence signatures emerge from integrated analysis

To investigate potential cellular senescence induction, we performed integrated analysis of cell cycle regulators and senescence-associated genes (**Figure 6**). Macrophages showed coordinated changes in multiple senescence markers, particularly in the G1 phase marker set, suggesting potential senescence-associated cell cycle arrest, and was further supported by the upregulation of known senescence-associated genes such as Cd81 and Nrp1. However, NK cells displayed no similar senescence-associated gene activation, correlating with their absence of significant cell cycle phase redistribution following PM2.5 exposure. These findings indicate cell type-specific senescence responses to PM2.5 exposure, with potential implications for immune function and tissue homeostasis.

## 4 Discussion

Our study reveals that PM2.5 exposure induces cell type-specific alterations in uterine immune cell populations and their cell cycle dynamics. Single-cell RNA sequencing demonstrated significant reductions in macrophages, granulocytes, and NK cells, which is highly consistent with our previous study (9). Cell cycle analysis uncovered distinct phase redistributions specifically G1 accumulation and G2M reduction in macrophages. These findings suggest that PM2.5 disrupts uterine immune homeostasis through reprogramming of cell cycle progression, with potential implications for reproductive health.

Our current approach prioritized immune cell populations with sufficient cell numbers to enable detailed cell cycle analysis, and this methodological choice was necessary to ensure statistical reliability in assessing cell cycle phase distributions and gene expression changes. The exclusion of smaller cell populations reflects a deliberate analytical strategy rather than a biological absence. This focus allowed us to uncover novel aspects of immune cell dysfunction that complement and extend previous findings in the field. While earlier studies by Xie et al. (13) and Lin et al. (14) reported general inflammatory responses to PM2.5 exposure, our analysis revealed cell type-specific alterations in cell cycle progression.

The observed changes in cell cycle distribution and gene expression patterns suggest complex mechanisms by which air pollution may impact immune function in the reproductive system. The most striking finding is the cell-type specific nature of the response to PM2.5 exposure, where the dramatic reduction in macrophage and NK cell populations aligns with previous studies showing PM2.5-induced immune cell redistribution (15, 16), but our study further reveals the underlying cell cycle mechanisms. The altered cell cycle distributions, particularly the decreased G1 phase in macrophages indicate disrupted cellular homeostasis that contributes to impaired immune function, which is highly consistent with previous studies (17).

The differential gene expression analysis revealed several key pathways that may mediate the effects of PM2.5 exposure. The upregulation of Cd81 and Nrp1 in macrophages suggests potential mechanisms for altered immune cell function and migration, consistent with findings from recent studies on various factors-induced macrophage dysfunction (18). The consistent changes in cell cycle-related genes across multiple cell types indicate that cell cycle disruption may be a common mechanism by which PM2.5 exposure affects immune cell function. Moreover, the observed changes in senescence-associated genes suggest that PM2.5 exposure may promote cellular senescence in specific immune cell populations, which could have important implications for tissue homeostasis and function, as senescent cells can contribute to chronic inflammation and tissue dysfunction through the senescence-associated secretory phenotype (SASP) (19).

Pathway enrichment analysis revealed distinct responses across immune cell types. While cell cycle pathway (enrichKEGG, p.adj = 0.79) and p53 signaling (gseKEGG, p.adj = 0.53) in NK cells did not meet strict significance thresholds (p.adj > 0.05), their inclusion reflects their biological relevance to cell cycle regulation and senescence. The gseKEGG analysis further highlighted subthreshold trends, such as Ribosome in macrophages (NES = 1.9, p.adj < 0.001), suggesting ribosomal dysfunction as a potential contributor to PM2.5-induced dysfunction. Together, these results underscore the value of combining enrichKEGG and gseKEGG to capture both thresholded and nuanced pathway-level changes.

Our study has several notable strengths and limitations that should be considered when interpreting the results. Key strengths include: 1) the application of single-cell RNA sequencing technology providing unprecedented resolution of cell-specific responses, 2) robust quality control measures and balanced sampling approach enhancing result reliability, and 3) comprehensive analysis of cell cycle dynamics across multiple immune cell populations. However, while cell cycle phase redistributions were observed, pathway enrichment analyses yielded inconsistent statistical significance for canonical cell cycle pathways across immune cell types. This discrepancy likely stems from methodological nuances inherent to single-cell transcriptomics. Specifically, cell cycle scoring captures phase-specific transcriptional shifts at the single-cell level, which may not always align with pathway-level enrichment thresholds designed for bulk RNA-seq. For instance, subtle but coordinated changes in cell cycle regulators may drive phase redistributions without reaching stringent pathway significance, particularly in smaller cell populations like dendritic cells. Additionally, the stringent multiple-testing corrections applied in pathway analysis may obscure biologically relevant trends, especially in heterogenous immune subsets. Besides, other limitations include the inability to track individual cells over time, potential loss of rare cell populations due to our stringent analytical strategy, and challenges in distinguishing direct PM2.5 effects from secondary responses. While our study focused on major immune populations to ensure statistical rigor, future investigations with larger sample sizes could elucidate subtype-specific responses (e.g., M1/M2 macrophage polarization or T cell subsets). Such analyses may reveal finer-grained mechanisms of PM2.5-induced immune dysregulation.

In conclusion, our study demonstrates that PM2.5 exposure induces distinct cell cycle alterations across different uterine immune cell populations. The cell type-specific nature of these responses, particularly the divergent cell cycle changes in macrophages versus NK cells, suggests complex mechanisms of PM2.5-induced immune dysfunction. These findings not only advance our understanding of environmental impacts on reproductive health but also identify potential therapeutic targets for preventing or mitigating PM2.5-related reproductive complications. Future studies should focus on validating these findings in human tissues and developing targeted interventions to protect reproductive function in polluted environments.

## Data Availability

The original contributions presented in the study are included in the article/**Supplementary Material**. Further inquiries can be directed to the corresponding author.
