# Supplementary material for: PM2.5 exposure reprograms cell cycle dynamics in uterine immune cells at single-cell resolution
Source: Front Immunol. 2025 Apr 1;16:1561290. doi: 10.3389/fimmu.2025.1561290 (PMC11996630; doi:10.3389/fimmu.2025.1561290)
Supplement: Supplementary file 1 [file DataSheet1.docx]

**Supplementary Tables**

**Table S1.** R packages with corresponding versions utilized in the analytical workflow

| **ID** | **Package** | **Version** | **ID** | **Package** | **Version** |
| --- | --- | --- | --- | --- | --- |
| 1 | cluster | 2.1.6 | 32 | BiocGenerics | 0.50.0 |
| 2 | DoubletFinder | 2.0.4 | 33 | Matrix | 1.7-1 |
| 3 | scplotter | 0.1.1 | 34 | lessR | 4.3.7 |
| 4 | GseaVis | 0.1.0 | 35 | ggtricks | 0.1.0 |
| 5 | pathview | 1.44.0 | 36 | hrbrthemes | 0.8.7 |
| 6 | Rgraphviz | 2.48.0 | 37 | viridis | 0.6.5 |
| 7 | topGO | 2.56.0 | 38 | viridisLite | 0.4.2 |
| 8 | SparseM | 1.84-2 | 39 | paletteer | 1.6.0 |
| 9 | GO.db | 3.19.1 | 40 | reshape2 | 1.4.4 |
| 10 | graph | 1.82.0 | 41 | scales | 1.3.0 |
| 11 | org.Mm.eg.db | 3.19.1 | 42 | RColorBrewer | 1.1-3 |
| 12 | AnnotationDbi | 1.66.0 | 43 | clipr | 0.8.0 |
| 13 | IRanges | 2.38.1 | 44 | xlsx | 0.6.5 |
| 14 | S4Vectors | 0.42.1 | 45 | readxl | 1.4.3 |
| 15 | clusterProfiler | 4.12.6 | 46 | harmony | 1.2.3 |
| 16 | rvcheck | 0.2.1 | 47 | Rcpp | 1.0.13-1 |
| 17 | eulerr | 7.0.2 | 48 | ggpubr | 0.6.0 |
| 18 | annosc | 0.1.0 | 49 | patchwork | 1.3.0 |
| 19 | circlize | 0.4.16 | 50 | lubridate | 1.9.3 |
| 20 | colorBlindness | 0.1.9 | 51 | forcats | 1.0.0 |
| 21 | ggthemr | 1.1.0 | 52 | stringr | 1.5.1 |
| 22 | ggsci | 3.2.0 | 53 | dplyr | 1.1.4 |
| 23 | ggthemes | 5.1.0 | 54 | purrr | 1.0.2 |
| 24 | ggSCvis | 0.0.2 | 55 | readr | 2.1.5 |
| 25 | scRNAtoolVis | 0.1.0 | 56 | tidyr | 1.3.1 |
| 26 | ClusterGVis | 0.1.1 | 57 | tibble | 3.2.1 |
| 27 | monocle | 2.32.0 | 58 | ggplot2 | 3.5.1 |
| 28 | DDRTree | 0.1.5 | 59 | tidyverse | 2.0.0 |
| 29 | irlba | 2.3.5.1 | 60 | Seurat | 5.1.0 |
| 30 | VGAM | 1.1-12 | 61 | SeuratObject | 5.0.2 |
| 31 | Biobase | 2.64.0 | 62 | sp | 2.1-4 |

**Table S2.** Top20 cell type-specific marker genes used for cluster annotation

| **ID** | **log2FC** | | **cluster** | | **gene** | | | **ID** | **log2FC** | | | **cluster** | | **gene** | |
| --- | --- | --- | --- | --- | --- | --- | --- | --- | --- | --- | --- | --- | --- | --- | --- |
| 1 | | 5.49 | | Fibr | | Mfap4 | 91 | | | 8.53 | NK | | Ms4a4b | |  |
| 2 | | 4.36 | | Fibr | | Sfrp2 | 92 | | | 9.51 | NK | | Trdc | |  |
| 3 | | 4.80 | | Fibr | | Pdgfra | 93 | | | 8.60 | NK | | Gimap7 | |  |
| 4 | | 5.13 | | Fibr | | Mfap5 | 94 | | | 8.45 | NK | | Podnl1 | |  |
| 5 | | 4.77 | | Fibr | | Steap3 | 95 | | | 8.39 | NK | | Cd226 | |  |
| 6 | | 4.44 | | Fibr | | Dpt | 96 | | | 9.17 | NK | | Sh2d2a | |  |
| 7 | | 4.51 | | Fibr | | Islr | 97 | | | 8.97 | NK | | Icos | |  |
| 8 | | 5.89 | | Fibr | | Aox3 | 98 | | | 9.00 | NK | | Tcrg-C1 | |  |
| 9 | | 4.91 | | Fibr | | Lsamp | 99 | | | 9.52 | NK | | Tcrg-C4 | |  |
| 10 | | 5.98 | | Fibr | | Wnt16 | 100 | | | 9.11 | NK | | Trdv4 | |  |
| 11 | | 4.97 | | Fibr | | A2m | 101 | | | 7.26 | Meso | | Msln | |  |
| 12 | | 4.29 | | Fibr | | Gdf10 | 102 | | | 7.20 | Meso | | Upk3b | |  |
| 13 | | 5.81 | | Fibr | | Scara5 | 103 | | | 7.44 | Meso | | Nkain4 | |  |
| 14 | | 4.28 | | Fibr | | Rasd2 | 104 | | | 7.74 | Meso | | Lgals2 | |  |
| 15 | | 4.25 | | Fibr | | Tex15 | 105 | | | 6.81 | Meso | | Prr15 | |  |
| 16 | | 4.86 | | Fibr | | Pappa | 106 | | | 6.91 | Meso | | Xpnpep2 | |  |
| 17 | | 4.22 | | Fibr | | Gm48898 | 107 | | | 7.75 | Meso | | Tm4sf5 | |  |
| 18 | | 5.57 | | Fibr | | D630033O11Rik | 108 | | | 8.88 | Meso | | Lrrn4 | |  |
| 19 | | 5.05 | | Fibr | | Hsd11b2 | 109 | | | 6.46 | Meso | | Fgf1 | |  |
| 20 | | 4.53 | | Fibr | | Wnt6 | 110 | | | 6.36 | Meso | | Fam180a | |  |
| 21 | | 7.31 | | Gran | | Il1b | 111 | | | 6.32 | Meso | | Cybrd1 | |  |
| 22 | | 7.92 | | Gran | | S100a8 | 112 | | | 6.89 | Meso | | Myrf | |  |
| 23 | | 7.55 | | Gran | | Csf3r | 113 | | | 6.55 | Meso | | Rab6b | |  |
| 24 | | 8.05 | | Gran | | S100a9 | 114 | | | 6.47 | Meso | | Folr1 | |  |
| 25 | | 7.67 | | Gran | | Hcar2 | 115 | | | 6.86 | Meso | | Rpp25 | |  |
| 26 | | 7.41 | | Gran | | G0s2 | 116 | | | 7.37 | Meso | | Muc16 | |  |
| 27 | | 8.23 | | Gran | | Cxcr2 | 117 | | | 6.28 | Meso | | Ildr2 | |  |
| 28 | | 7.40 | | Gran | | Acod1 | 118 | | | 7.82 | Meso | | Bnc1 | |  |
| 29 | | 7.59 | | Gran | | Gm13822 | 119 | | | 6.56 | Meso | | Pcnx2 | |  |
| 30 | | 7.50 | | Gran | | Clec4e | 120 | | | 6.80 | Meso | | Rprml | |  |
| 31 | | 7.31 | | Gran | | Mirt1 | 121 | | | 7.91 | Endo | | Egfl7 | |  |
| 32 | | 7.27 | | Gran | | Hp | 122 | | | 8.11 | Endo | | C130074G19Rik | |  |
| 33 | | 7.99 | | Gran | | Il1f9 | 123 | | | 9.54 | Endo | | Ptprb | |  |
| 34 | | 7.90 | | Gran | | Trem1 | 124 | | | 7.97 | Endo | | Emcn | |  |
| 35 | | 7.02 | | Gran | | Cd33 | 125 | | | 7.89 | Endo | | Abcb1a | |  |
| 36 | | 8.00 | | Gran | | Gm39321 | 126 | | | 9.06 | Endo | | Adgrl4 | |  |
| 37 | | 7.52 | | Gran | | Asprv1 | 127 | | | 8.70 | Endo | | Tie1 | |  |
| 38 | | 7.49 | | Gran | | Mirt2 | 128 | | | 8.75 | Endo | | Mmrn2 | |  |
| 39 | | 8.80 | | Gran | | Stfa2l1 | 129 | | | 8.85 | Endo | | Ushbp1 | |  |
| 40 | | 7.42 | | Gran | | Wfdc21 | 130 | | | 10.23 | Endo | | Cyyr1 | |  |
| 41 | | 6.08 | | Epit | | Cldn7 | 131 | | | 8.87 | Endo | | Myct1 | |  |
| 42 | | 5.94 | | Epit | | Wfdc2 | 132 | | | 8.31 | Endo | | Fam167b | |  |
| 43 | | 5.90 | | Epit | | Msx1 | 133 | | | 9.36 | Endo | | Sox18 | |  |
| 44 | | 6.09 | | Epit | | BC048679 | 134 | | | 9.69 | Endo | | Robo4 | |  |
| 45 | | 6.27 | | Epit | | Cldn3 | 135 | | | 9.76 | Endo | | Syt15 | |  |
| 46 | | 5.90 | | Epit | | Elf3 | 136 | | | 10.96 | Endo | | Aplnr | |  |
| 47 | | 6.76 | | Epit | | Fermt1 | 137 | | | 8.49 | Endo | | Tmem252 | |  |
| 48 | | 6.41 | | Epit | | Dlx5 | 138 | | | 9.12 | Endo | | Erg | |  |
| 49 | | 6.44 | | Epit | | 4833423E24Rik | 139 | | | 9.77 | Endo | | Gpihbp1 | |  |
| 50 | | 5.94 | | Epit | | Cldn4 | 140 | | | 8.30 | Endo | | Gm32688 | |  |
| 51 | | 5.91 | | Epit | | Fcgbp | 141 | | | 9.26 | Peri | | Rgs5 | |  |
| 52 | | 5.93 | | Epit | | Sftpd | 142 | | | 7.62 | Peri | | Ndufa4l2 | |  |
| 53 | | 6.23 | | Epit | | S100g | 143 | | | 8.59 | Peri | | Higd1b | |  |
| 54 | | 6.34 | | Epit | | Prap1 | 144 | | | 6.53 | Peri | | Mustn1 | |  |
| 55 | | 6.25 | | Epit | | Pax8 | 145 | | | 7.72 | Peri | | Notch3 | |  |
| 56 | | 6.36 | | Epit | | Clca1 | 146 | | | 6.46 | Peri | | Kcnj8 | |  |
| 57 | | 6.07 | | Epit | | St14 | 147 | | | 7.25 | Peri | | Gucy1b1 | |  |
| 58 | | 6.04 | | Epit | | Urah | 148 | | | 5.90 | Peri | | Cspg4 | |  |
| 59 | | 6.97 | | Epit | | Tmem45b | 149 | | | 7.51 | Peri | | Abcc9 | |  |
| 60 | | 6.36 | | Epit | | Tspan1 | 150 | | | 8.06 | Peri | | Rgs4 | |  |
| 61 | | 6.93 | | Macr | | Cd74 | 151 | | | 6.92 | Peri | | Kcnk3 | |  |
| 62 | | 6.75 | | Macr | | Aif1 | 152 | | | 8.74 | Peri | | Olfr558 | |  |
| 63 | | 7.53 | | Macr | | Ms4a7 | 153 | | | 5.80 | Peri | | Ednrb | |  |
| 64 | | 7.55 | | Macr | | Pld4 | 154 | | | 5.88 | Peri | | Gja4 | |  |
| 65 | | 7.48 | | Macr | | Trem2 | 155 | | | 6.38 | Peri | | Slc7a2 | |  |
| 66 | | 7.03 | | Macr | | H2-Aa | 156 | | | 7.65 | Peri | | Foxs1 | |  |
| 67 | | 7.34 | | Macr | | Ms4a6c | 157 | | | 8.98 | Peri | | Vtn | |  |
| 68 | | 7.60 | | Macr | | C1qc | 158 | | | 5.72 | Peri | | Olfml2b | |  |
| 69 | | 7.54 | | Macr | | C1qb | 159 | | | 6.67 | Peri | | Heyl | |  |
| 70 | | 6.59 | | Macr | | Ifi207 | 160 | | | 6.47 | Peri | | Rasl12 | |  |
| 71 | | 7.80 | | Macr | | C1qa | 161 | | | 6.61 | SMC | | Tagln | |  |
| 72 | | 6.86 | | Macr | | H2-Eb1 | 162 | | | 5.84 | SMC | | Acta2 | |  |
| 73 | | 7.61 | | Macr | | Ly86 | 163 | | | 7.31 | SMC | | Myh11 | |  |
| 74 | | 7.22 | | Macr | | H2-Ea-ps | 164 | | | 8.86 | SMC | | Pcp4 | |  |
| 75 | | 7.22 | | Macr | | Ms4a6d | 165 | | | 5.28 | SMC | | Smtn | |  |
| 76 | | 7.43 | | Macr | | C3ar1 | 166 | | | 7.74 | SMC | | Cnn1 | |  |
| 77 | | 6.46 | | Macr | | Pf4 | 167 | | | 9.23 | SMC | | Actg2 | |  |
| 78 | | 8.47 | | Macr | | Cx3cr1 | 168 | | | 7.28 | SMC | | Lmod1 | |  |
| 79 | | 6.86 | | Macr | | Adgre1 | 169 | | | 6.08 | SMC | | Fbxl22 | |  |
| 80 | | 6.43 | | Macr | | Ms4a4c | 170 | | | 6.69 | SMC | | Sh3bgr | |  |
| 81 | | 8.67 | | NK | | Cd3g | 171 | | | 5.63 | SMC | | Aspn | |  |
| 82 | | 8.46 | | NK | | Trbc2 | 172 | | | 6.84 | SMC | | Hmcn2 | |  |
| 83 | | 8.93 | | NK | | Lat | 173 | | | 6.06 | SMC | | Jph2 | |  |
| 84 | | 9.76 | | NK | | Cxcr6 | 174 | | | 7.15 | SMC | | Syndig1 | |  |
| 85 | | 9.20 | | NK | | Trbc1 | 175 | | | 6.25 | SMC | | Kcnh2 | |  |
| 86 | | 8.49 | | NK | | Gimap3 | 176 | | | 7.45 | SMC | | Aard | |  |
| 87 | | 11.51 | | NK | | Nkg7 | 177 | | | 7.41 | SMC | | Tnnt2 | |  |
| 88 | | 9.32 | | NK | | Il2rb | 178 | | | 5.30 | SMC | | Aoc3 | |  |
| 89 | | 8.58 | | NK | | Ctsw | 179 | | | 5.25 | SMC | | Rspo3 | |  |
| 90 | | 8.65 | | NK | | Skap1 | 180 | | | 5.41 | SMC | | Cacnb2 | |  |

**Supplementary Figures**


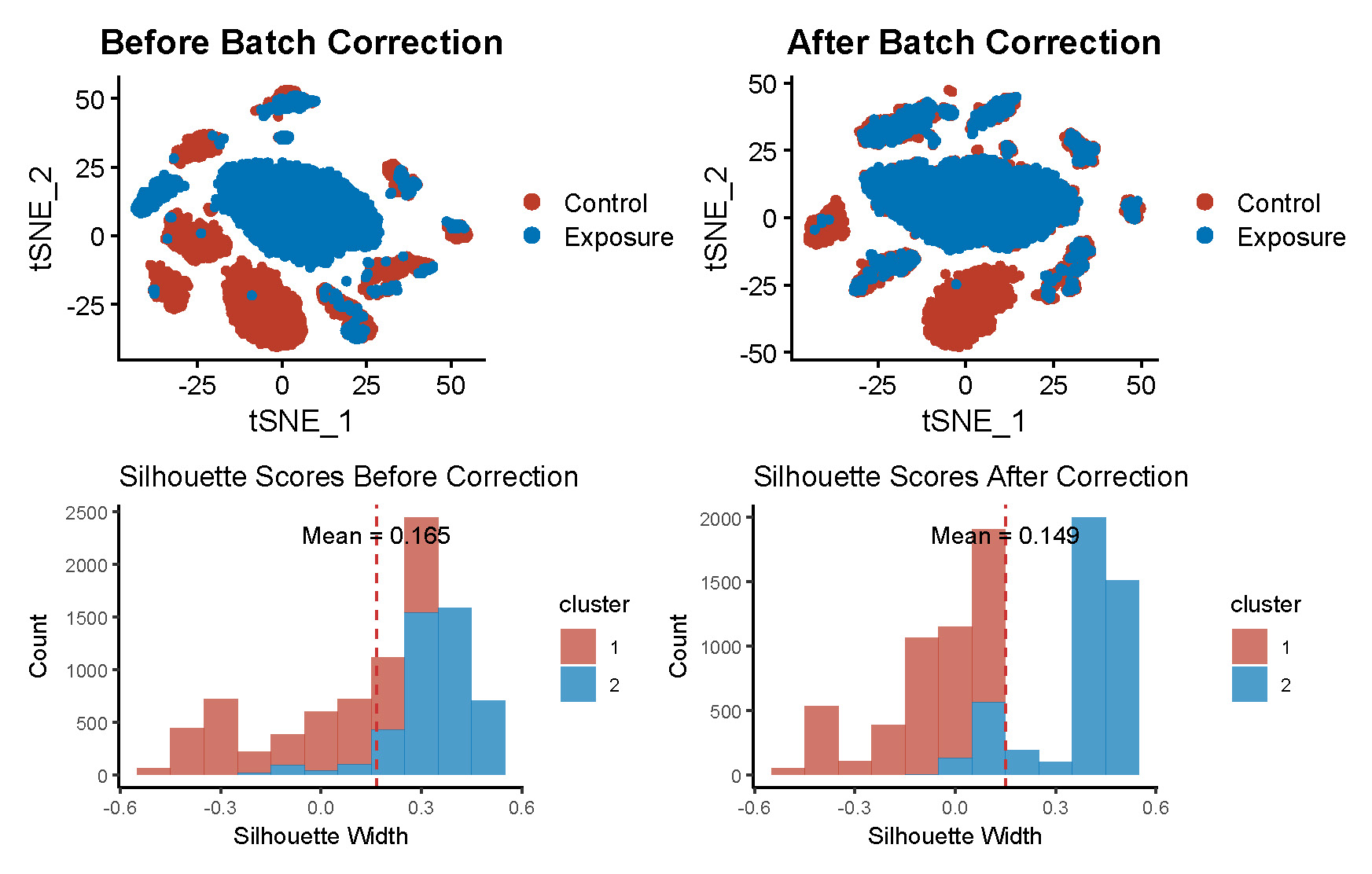


**Figure S1.** Batch effect evaluation and correction for scRNA-seq data
